# Remote sensing of geomagnetic fields and atomic collisions in the mesosphere

**DOI:** 10.1038/s41467-018-06396-7

**Published:** 2018-09-28

**Authors:** Felipe Pedreros Bustos, Domenico Bonaccini Calia, Dmitry Budker, Mauro Centrone, Joschua Hellemeier, Paul Hickson, Ronald Holzlöhner, Simon Rochester

**Affiliations:** 1 Helmholtz Institute Mainz, Johannes Gutenberg University, Staudingerweg 18, 55128 Mainz, Germany; 2 European Southern Observatory, Karl-Schwarzschild-Str. 2, 85748 Garching bei München, Germany; 3 Department of Physics, University of California Berkeley, Berkeley, CA 94720-7300 USA; 4 Istituto Nazionale di Astrofisica, Osservatorio Astronomico di Roma, Via Frascati, 33, 00078 Monte Porzio Catone, RM Italy; 5 Department of Physics and Astronomy, University of British Columbia, 6224 Agricultural Road, Vancouver, BC V6T1Z1 Canada; 6 Rochester Scientific LLC, 2041 Tapscott Ave., El Cerrito, 94530 CA USA

## Abstract

Magnetic-field sensing has contributed to the formulation of the plate-tectonics theory, mapping of underground structures on Earth, and the study of magnetism of other planets. Filling the gap between space-based and near-Earth observations, we demonstrate a remote measurement of the geomagnetic field at an altitude of 85–100 km. The method consists of optical pumping of atomic sodium in the mesosphere with an intensity-modulated laser beam, and ground-based observation of the resultant magneto-optical resonance near the Larmor precession frequency. Here we validate this technique and measure the Larmor precession frequency of sodium and the corresponding magnetic field with an accuracy level of 0.28 mG Hz^−1/2^. These observations allow the characterization of atomic-collision processes in the mesosphere. Remote detection of mesospheric magnetic fields has potential applications such as mapping magnetic structures in the lithosphere, monitoring space weather, and electric currents in the ionosphere.

## Abstract (web summary)

Remote sensing of geomagnetic fields in mesosphere is both challenging and interesting to explore the magnetic field structures and atomic collision processes. Here the authors demonstrate an atomic magnetometer that utilizes the Larmor frequency in sodium atoms and operates in kilometers range.

## Introduction

Laser excitation of the atomic sodium layer, located between 85 and 100 km altitude in the upper mesosphere, allows astronomers to create artificial light sources, known as Laser Guide Stars (LGS), to assist adaptive optics systems^1^. A laser beam tuned to a wavelength resonant with the 3S_1/2_ → 3P_3/2_ transition in sodium produces atomic fluorescence that is collected at ground with a telescope for real-time compensation of atmospheric turbulence in astronomical observations. Since the introduction of this technique^2,3^, research has been conducted to optimize laser excitation schemes in order to maximize the flux of photons returned to the ground. This technological progress has also catalyzed new concepts of laser remote sensing of magnetic fields with mesospheric sodium^4^. Because of the proximity of the sodium layer to the D and E regions of the ionosphere (between 70 and 120 km altitude) mesospheric magnetometry opens the possibility to map local current structures in the dynamo region^5,6^. In addition, the capability of continuously monitoring the geomagnetic field at altitudes of 85–100 km could provide valuable information for modeling the geomagnetic field, detection of oceanic currents^7^, and for mapping and identification of large-scale magnetic structures in the upper mantle^8^.

In a laser magnetometer, atoms are optically polarized and the effects of the interaction of the polarized atoms with magnetic fields are observed^9^. For instance, optical pumping of sodium with left-hand circularly polarized light produces atomic polarization in the $\left| {F = 2} \right\rangle$ ground state (*F* is the total angular momentum quantum number), which is continuously depolarized as the angular momentum precesses at the Larmor frequency in the local magnetic field^10^. If the medium is pumped with light pulses synchronized with the Larmor precession, a high degree of atomic polarization can be obtained and an increase in the fluorescence in the cycling transition $\left| {F = 2} \right\rangle$ ↔ $\left| {F\prime = 3} \right\rangle$ can be observed (the prime refers to the excited atomic state)^11^. The direct measurement of the Lamor frequency (*f*_Larmor_) gives the magnetic field *B* from

1 $$f_{{\mathrm{Larmor}}} = \gamma B,$$

where *γ* is the gyromagnetic ratio of ground-state sodium given by *γ* = 699,812 Hz G^−1^. This relationship applies to weak magnetic fields where Zeeman splitting of the energy levels depends linearly on the field. Therefore, as proposed in ref.^4^, pumping the sodium layer with an intensity-modulated laser beam and observing the magneto-optical resonance occurring at the Lamor frequency from the surface of the Earth allows one to remotely detect the magnetic field in the mesosphere. The first observation of a magnetic resonance and remote magnetic field determination in the mesosphere were recently reported in ref. ^12^ Here, we demonstrate mesospheric magnetometry with an order-of-magnitude better sensitivity due to a number of factors, including exploiting the narrower magnetic resonance feature. In this work, the observed characteristic spectroscopic features of the resonance curve enable quantitative characterization of collisional processes in the mesosphere.

In the following, we describe the details of our experiment to measure the Larmor precision frequency of sodium atoms. We discuss the resonance features, precise measurements possible with our magnetometry method and the collision relaxation rates. In addition to magnetometry, our observations have yielded quantitative information about collisional processes in the mesosphere, which is important for the optimization of sodium laser guide stars and mesospheric magnetometers.

## Results

### Experimental arrangement

**Fig. 1:**
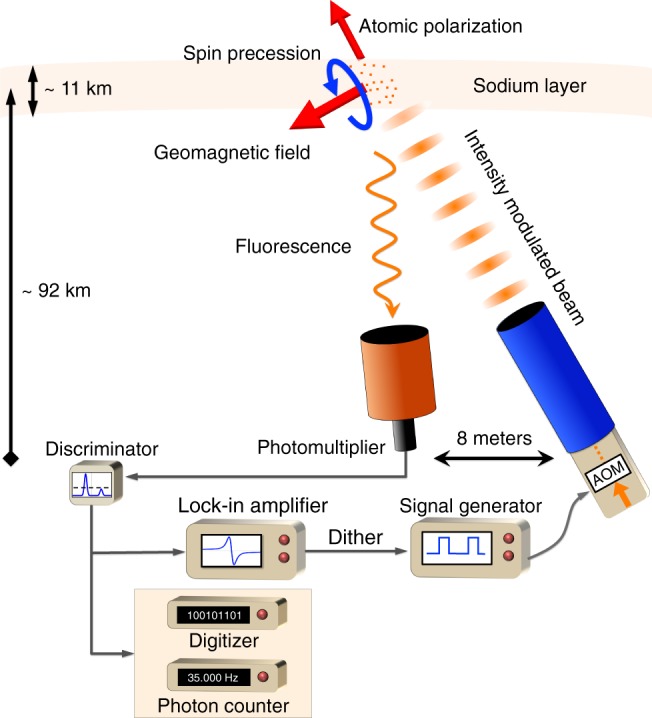
Experimental arrangement. A laser projector sends an intensity-modulated beam to the mesosphere where it polarizes sodium atoms. Fluorescence is observed with a second telescope and the received photons are recorded, counted and demodulated with a digitizer, a photon counter, and a lock-in amplifier, respectively. The change in fluorescence is measured as the laser modulation frequency is swept around the Larmor frequency driving the acousto-optic modulator (AOM) using a signal generator. The lock-in amplifier provides the reference to dither the intensity-modulation frequency to discriminate atmospheric scintillation noise

The experimental setup is depicted in Fig. 1. It used the European Southern Observatory Wendelstein Laser Guide Star Unit (ESO WLGSU) installed next to the William Herschel Telescope (WHT) at the Observatorio del Roque de los Muchachos (ORM) in La Palma (Supplementary Fig. 1). The operation of the WLGSU allows modulation of the beam and pointing the transmitter and receiver telescopes at the same target. The setup incorporated a laser projector telescope and a receiver telescope separated by eight meters. The light source consisted of a continuous-wave Raman-fiber-amplified frequency-doubled laser with a maximum output power of 20 W^13^. The laser was tuned to the vacuum wavelength of 589.158 nm corresponding to the 3S_1/2_ → 3P_3/2_ transition of sodium (the D2 line); the linewidth of the laser was measured to be ~2 MHz. The laser system incorporated an AOM (acousto-optic modulator) for on-off amplitude modulation of the beam intensity. The beam polarization was controlled with a set of waveplates following the AOM. The Galilean projector telescope magnified the beam to an output diameter of 30 cm. The receiver consisted of a 40-cm aperture Schmidt–Cassegrain telescope mounted on the WLGSU receiver control unit, equipped with a narrow-band interference filter of 0.30(5) nm bandwidth centered at the sodium D2 line wavelength, a tracking CMOS (complementary metal-oxide-semiconductor) camera and a PMT (photomultiplier tube). A discriminator was used to filter and convert the analog pulses from the PMT into 100 ns TTL (transistor–transistor logic) pulses for the photon counters. The signal was acquired by three independent methods: (a) digitizing and counting the arrival of individual photons (offline mode), (b) directly measuring and averaging the photon-count difference per modulation period (online counter), and (c) directly demodulating the signal from the PMT with a lock-in amplifier.

Each observation run consisted of a discrete frequency sweep of the laser intensity modulation around the predicted Larmor frequency. In order to reduce atmospheric scintillation noise, the frequency of the laser intensity modulation at each step of the sweep (*f*_step_) was dithered with a square-wave function such that

2 $$f_{{\mathrm{pulse}}}(t) = f_{{\mathrm{step}}} + \delta f \cdot {\mathrm{sgn}}\left[ {{\mathrm{cos}}\left( {2\pi f_{\mathrm{m}}t} \right)} \right],$$

where *f*_pulse_(*t*) is the frequency of the laser intensity modulation, *δf* is the excursion of the dither, *f*_m_ is the dither frequency and sgn is the sign function. The information of the magneto-optical resonance is therefore contained in the amplitude of the alternating signal which oscillates at *f*_m_. When *f*_step_ increases and approaches the magneto-optical resonance, a dip (or peak) occurs depending on the polarity of the reference signal used for demodulation. The opposite situation occurs when the intensity-modulation frequency exceeds the Larmor frequency along the sweep. Therefore, demodulation produces a peak and a dip separated from each other by 2*δf* and centered at *f*_Larmor_. The excursion was varied from *δf* = 8–45 kHz to find the optimal separation between demodulated peaks, and the dither frequency was fixed at *f*_m_ = 150 Hz to suppress scintillation noise.

Because some of the sodium atoms decay into the dark *F* = 1 ground state^14^, a fraction of the laser power (12%) was detuned by +1.713 GHz in order to maximize the number of available atoms by pumping them back into the *F* = 2 ground state via the *F*′ = 2 excited state.

The duty cycle of the laser intensity modulation was varied from 10 to 30%, as a compromise between high return flux and effective optical pumping. Laser polarization was kept circular for all runs in order to prepare the required orientation of the atomic spins along the laser-beam direction. The laser beam pointed in a direction at which the magnetic field vector in the mesosphere was approximately perpendicular to the laser-beam axis, which gives the highest contrast for the magneto-optical resonance. According to the World Magnetic Model (WMM2015)^15^, the declination and inclination of the magnetic field at La Palma are 5.7° West and 39.1° downwards, respectively. Therefore, observations were carried out at an elevation of about 51° in the northern direction. Nevertheless, pointing at higher elevation up to 75° was also explored in order to reduce the airmass contribution to scintillation and the magnetic field uncertainty due to a shorter sodium layer path along the laser beam. From the WMM2015, the estimated magnetic field strength at 92 km altitude is 0.3735(15) G, corresponding to a predicted Larmor frequency of ~261 kHz.

The duration of each run depended on the frequency range of the sweep and the integration time for each step. About 10 minutes were necessary to perform a sweep of ±75 kHz around the Larmor frequency. During five nights of observations from July 2nd to July 6th 2017, there were 51 successful runs. Laser power, duty cycle and excursion parameters were modified from run to run to investigate their effects on the magneto-optical resonance. The average atmospheric seeing was 0.7 arcsec measured at zenith and at 500 nm, as reported by a seeing Differential Image Motion Monitor (DIMM) collocated at the observatory. Data from the seeing monitor are available online from the website of the Isaac Newton Group of Telescopes (ING)^16^.

**Fig. 2:**
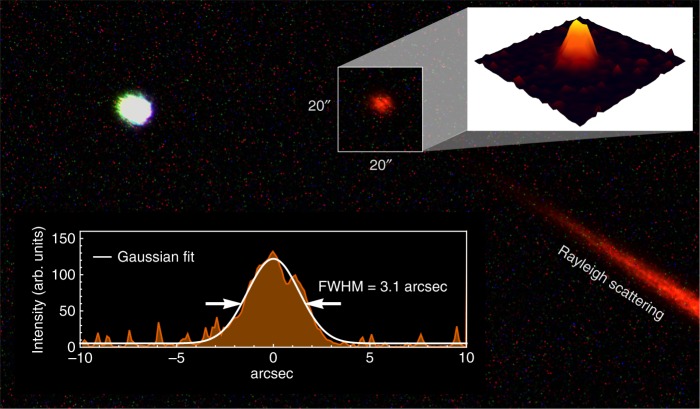
Fluorescence of mesospheric sodium. A five-second-exposure raw image of the sodium fluorescence spot in the mesosphere along with the star HIP113889 obtained with the CMOS camera of the receiver telescope. The estimated long-term spot size is 3.1 arcsec, which comprises broadening due to atmospheric propagation and focusing error of the receiver telescope. Rayleigh scattering from the laser propagation in lower layers of the atmosphere is visible in the bottom-right corner of the image

Physical-optics modeling of the mesospheric spot size under these conditions gives an instantaneous full-width-at-half-maximum (FWHM) beam diamater of *D*_FWHM_ = 36 cm (0.8 arcsec) for a 30-cm launch telescope at an elevation angle of *θ*_EL_ = 60°, and average mesospheric irradiance of $I_{{\mathrm{avg}}}^{{\mathrm{meso}}} = 15$ W m^−2^ for 2-W CW output power (because of the duty cycle and finite AOM efficiency, 10–20% of the average laser power was delivered to the sky). The spot size in the mesosphere was estimated from a long-exposure image taken with the receiver CMOS camera to be 3.1 arcsec (Fig. 2), however, this estimate is subject to the effects of double-pass laser propagation through the atmosphere, beam-wander, and focusing error of the receiver, each of which contribute to broaden the apparent fluorescent spot beyond the instantaneous spot size. Since the spin-precession dynamics occurs on time scales of microseconds, we calculate the irradiance in the mesosphere using the instantaneous beam size obtained from physical-optics^17^.

### Magneto-optical resonances

**Fig. 3:**
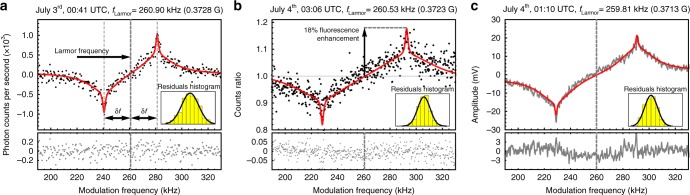
Magneto-optical resonances. The resonances were obtained by sweeping the frequency of the intensity-modulated laser beam with three concurrent data acquisition methods. **a** Online differential counter for a modulation duty cycle of 20% and $I_{{\mathrm{avg}}}^{{\mathrm{meso}}} = 13$ W m^−2^. The Larmor frequency lies in the center between the peaks, which are separated by twice the dither excursion *δf* = 20.2 kHz. **b** Ratio of the photon counts per dither period averaged over 2 s. The modulation duty cycle was 30%, excursion *δf* = 30.8 kHz and calculated mesospheric irradiance $I_{{\mathrm{avg}}}^{{\mathrm{meso}}} = 33$ W m^−2^. **c** Lock-in amplifier with time constant of 300 ms, modulation duty cycle 20%, excursion *δf* = 30.8 kHz, and calculated mesospheric irradiance $I_{{\mathrm{avg}}}^{{\mathrm{meso}}} = 17$ W m^−2^. For all resonances, a double Lorentzian fit shows a broad and a narrow width of ~30 and 2 kHz, respectively, consistent with two relaxation mechanisms due to velocity-changing collisions (fast) and spin-exchange collisions (slow) of sodium with N_2_ and O_2_ molecules. The residuals of the fits are shown below each resonance and obey a normal distribution according to the Gaussian fit of the residuals histograms

Figure 3 shows three typical demodulated signals obtained with an online differential counter (Fig. 3a), an offline ratio counter (Fig. 3b), and a lock-in amplifier (Fig. 3c). The online counter reported the real-time difference in the photon counts between two half-periods of the dither signal, averaged over the time of each frequency step (2–3 s). The averaged maximum count difference per dither period of 6.7 ms (150 Hz) was only about seven photon counts, when the frequency reached the Larmor frequency. Therefore, the maximum averaged difference between off-resonance and on-resonance is about 1000 counts s^−1^ as shown in Fig. 3a. A higher dither frequency would have rejected scintillation noise better, at the cost of fewer photon counts per dither period. The digitizer recorded all photon counts and the ratio between alternating dither sub-periods was calculated. During post-processing, the phase of the square-wave dither signal could be freely adjusted. This is in contrast to the case of the online counter, where a wrong input phase could suppress the signal without the possibility of recovering it in post-processing. The enhancement in fluorescence of the excited sodium atoms when modulating in resonance with the Larmor precession (referred to as contrast) was measured as 18% above the photon flux out of resonance as shown in Fig. 3b. In addition, the lock-in amplifier demodulated the incoming signal into phase and quadrature components, calculating in real time the time-evolution of the resonance, useful for tracking slowly varying magnetic signals. A time constant of 300 ms was used for all measurements with lock-in amplifier.

The demodulated signals, consisting of a positive and a negative peak, were fit with superimposed Lorentzians (Fig. 3), following the outcome of a numerical model which is discussed below. The Larmor frequency was estimated as the mid-point between the two peaks. The residuals from the lock-in amplifier signal display small deviations from the fit that may be attributed  to slow altitude displacements of the sodium layer centroid during the sweep. Upward displacement of the sodium centroid toward a weaker magnetic field region produces a shift of the magnetic resonance toward lower frequencies, resulting in asymmetries of the observed resonance.

**Fig. 4:**
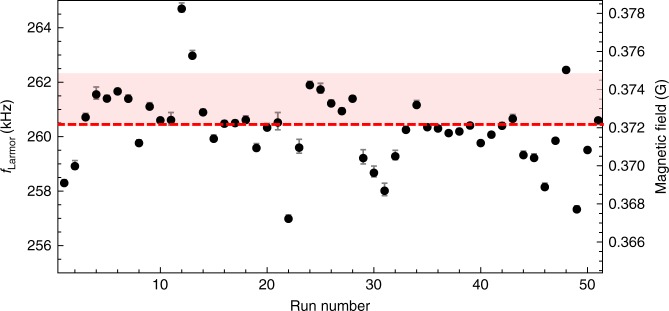
Measured Larmor frequency from 51 runs. The red dashed line is the median  of all observations. The horizontal light-red band represents the predicted magnetic field between 85 and 100 km altitude according to the WMM2015 magnetic model^15^. Error bars are the standard error of the estimate of *f*_Larmor_

The measured Larmor frequencies from 51 runs are plotted in Fig. 4. The average Larmor frequency was found to be 260.4(1) kHz, representing a geomagnetic field of 0.3720(1) G according to Eq. (1). The WMM2015 prediction for the magnetic field at 92 km altitude is 0.3735(15) G, giving a difference of <0.5% between the model and our observations. Since the magneto-optical signal comprises the contribution from all sodium atoms weighted by their density distribution along the laser interrogated column in the mesosphere, the measured Larmor frequency is most strongly representative of the geomagnetic field at the sodium centroid position. Indeed, due to magnetic field gradients in the vertical direction *H* in the mesosphere of d*B*/d*H* = −1.85 × 10^−4^ G km^−1^^15^, equivalent to a Larmor frequency gradient of d*f*_Larmor_/d*H* = −0.129 kHz km^−1^, the position of the Larmor frequency in the magneto-optical resonance could lie at any point within the light-red band shown in Fig. 4, depending on the position of the sodium centroid at the time of the observation.

In addition, spatially separated sodium density peaks (sporadic sodium layers)^18^ broaden the magneto-optical resonance as a result of atomic spins precessing at different Larmor frequencies due to magnetic field gradients within the sodium layer. Sporadic sodium layers in the mesosphere at La Palma have been detected on average once per night with lifetime from 30 s to several hours^19^, which makes our technique susceptible to this effect. The spatial accuracy of the magnetic-field measurements could be improved if the vertical sodium profile were independently known, for example, from simultaneous lidar (light detection and ranging) measurements. Because of the absence of such profiles during the present experiment, there is an intrinsic uncertainty in the altitude of the magnetic-field measurements.

### Magnetometry

To measure the absolute magnetic field in the mesosphere, a full scan of the magneto-optical resonance was performed so that the Larmor frequency could be determined. If it is desired to measure fluctuations in the magnetic field, the magnetometer can operate with an intensity-modulation frequency fixed at the maximum-sensitivity point along the resonance curve. In this case, magnetic-field variations are reflected in changes of the amplitude of the demodulated signal or in changes of the frequency feedback signal needed to keep the magnetometer locked at a certain point of the resonance curve.

**Fig. 5:**
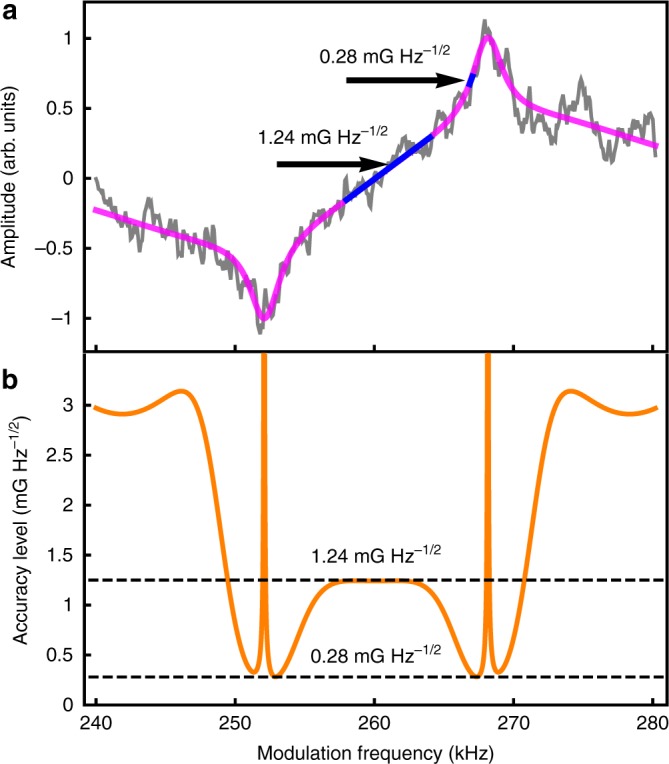
Estimate of magnetometry accuracy. **a** A resonance acquired with the lock-in amplifier in good atmospheric conditions (seeing 0.7 arcsec) with *δf* = 8 kHz. **b** Magnetometry accuracy level from the derivative of the resonance fit function. The maximum sensitivity of 0.28 mG Hz^−1/2^ is achieved at the steepest points of the resonance

In order to estimate the accuracy of the Larmor-frequency measurements and magnetic-field fluctuations, we use data from a single run with *δf* = 8 kHz and *f*_m_ = 150 Hz, as shown in Fig. 5a. The Larmor frequency for this run is 260.12 kHz, corresponding  to 0.37170 G, with a standard error for *f*_Larmor_ of 0.04 kHz (or 0.05 mG). The existence of the narrow peaks in the magneto-optical resonances found in this experiment strongly reduce the uncertainty in the estimate of the Larmor frequency. The highest magnetic-field sensitivity can be found at the minimum of the differentiated fit function of the resonance in Fig. 5b. At the middle point between the two peaks of the reference magneto-optical resonance shown in Fig. 5a, the calculated accuracy is 1.24 mG Hz^−1/2^, similar to that reported in ref. ^12^ The highest sensitivity is provided by the slope of the narrower of the two superimposed Lorentzians, where an accuracy of 0.28 mG Hz^−1/2^ can be reached.

In this experiment, a median value of about 12 × 10^3^ counts s^−1^ was measured during frequency scans, which corresponds to shot noise near 100 counts s^−1^ or ~1%. The estimate of the noise contributions from the noise analysis (Methods Section and Supplementary Fig. 2) shows a noise floor near 10^−2^ Hz^−1/2^, indicating a shot-noise-limited measurement. Random fluctuations of the centroid and the sodium layer profile are strong contributors to the uncertainty in the estimation of the geomagnetic field at a given point in the sodium layer.

## Discussion

The fundamental sensitivity of an optical magnetometer is determined by the total number of atoms, the spin-relaxation rate in the atomic medium, and the measurement duration^20^. At ORM, an average column density of *C*_*n*_ = 3.6 × 10^13^ atoms m^−2^ was measured with lidar observations^19^. From long-term observations of the sodium layer at low geographic latitude, the average sodium centroid height was determined to be 92 km above sea level with a thickness of 11.3 km^21^. The spin relaxation in the mesosphere is dominated by collisions and the finite transit time of the polarized atoms across the laser beam. For a sodium atom, most collisions occur with N_2_ and O_2_ molecules, whereas Na–Na collisions are less frequent due to the low sodium density. While collisions of sodium with any molecule change the velocity of the atoms, Na–O_2_ collisions are primarily responsible for spin relaxation due to the large exchange interaction between unpaired electrons of O_2_ and sodium^22^. The Na–O_2_ collisions determine the highest spin-relaxation rate in the atomic system [on the order of 1/(250 μs)] and limit the sensitivity to magnetic-field measurements by broadening the magneto-optical resonance. In the mesosphere, the diffusive transit of sodium atoms across the laser beam is expected to be one order of magnitude longer than the relaxation time given by Na–O_2_ collisions. Considering the aforementioned values of sodium density and spin-relaxation rate, the fundamental spin-projection-noise-limited sensitivity (quantum limit) is on the order of 10^−11^ G Hz^−1/2^. However, primarily due to the small solid angle for the fluorescence collection (~10^−11^ sr for a 0.4 m diameter receiver telescope), a shot-noise-limited sensitivity on the order of 10^−4^ G Hz^−1/2^ can be achieved.

The sensitivity can be affected by instabilities of the sodium layer. The sodium atomic density in the mesosphere is highly variable on all relevant time scales. Continuous monitoring of the sodium-layer density profiles with lidar techniques shows structural and density changes with time scales of minutes^23^. Sporadic events caused by the advection of meteor ablation from the ionosphere into the mesosphere produce sodium density changes over time scales of seconds^24^. In addition, atmospheric scintillation imposes another strong source of noise for an optical magnetometer. The power spectrum of scintillation was characterized at La Palma and shows a steep decrease for frequencies above 10 Hz for telescope apertures similar to those used in our experiment^25^. We have reached a sensitivity near the shot-noise limit, meaning that the approach of dithering the intensity modulation of the laser at a frequency of 150 Hz effectively removed most of the  intensity noise due to scintillation.

**Fig. 6:**
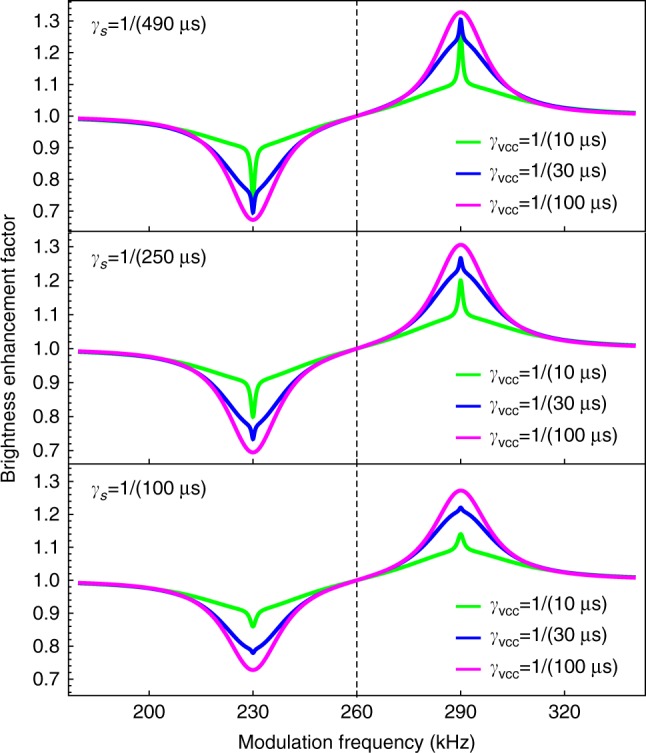
Numerical modeling of the magneto-optical resonance. We assume a laser irradiance of $I_{{\mathrm{avg}}}^{{\mathrm{meso}}} = 15$ W m^−2^ in the mesosphere, and excursion of *δf* = 30 kHz. The central dashed line indicates the Larmor frequency, *γ*_vcc_ denotes velocity-changing collision rates, and *γ*_s_ spin-exchange collision rates

Numerical modeling of the time evolution of the sodium atomic polarization under resonant pulsed excitation using the density-matrix model described in ref. ^26^ shows a magnetic resonance that can be fit with two superimposed Lorentzians of different widths. The superimposed Lorentzians (see also ref. ^27^) are analogous to the nested dispersive Lorentzians observed in nonlinear magneto-optical rotation (NMOR) with antirelaxation-coated vapor cells^28^. In such NMOR experiments, a transit effect is observed with a resonance width corresponding to the rate at which atoms traverse the light beam.  Moreover, a wall effect due to atoms leaving and then reentering the light beam after bouncing off of the cell wall, shows a narrower width corresponding to the relaxation rate of atomic ground-state polarization. In the present case, rather than considering the atomic positions relative to the light beam, we must consider atoms leaving and reentering the resonant velocity group of the Doppler distribution. Then, the broad resonance arises from precessing atoms leaving the resonant velocity class due to velocity-changing collisions (a type of transit effect within the Doppler distribution). On the other hand, the narrower resonance is determined by the polarization relaxation rate due to spin-exchange collisions in all velocity groups. The width of each feature equals 1/(*πτ*), where *τ* is the corresponding relaxation time. The effect of varying the rate of velocity-changing (*γ*_vcc_) and spin-exchange (*γ*_s_) collisions is shown in simulated resonance curves obtained from our model (Fig. 6).

Fitting experimental data with a double Lorentzian function and estimating the widths yields FWHM median values of Δ*f*_broad_ = 32 kHz for the broad resonance component, and Δ*f*_narrow_ = 2.4 kHz for the narrow resonance component. According to our numerical simulations the observed widths are obtained with a velocity-changing collision rate on the order of *γ*_vcc_ ≈ 1/(10 μs) and a spin-exchange rate on the order of *γ*_s_ ≈ 1/(100 μs). These results suggest that collision rates in the mesosphere are higher than previous estimates by a factor of 2–6. For instance, a mean spin-exchange collision rate of *γ*_s_ = 1/(490 μs) was estimated in ref. ^29^ Other estimates suggest values of *γ*_s_ ≈ 1/(200 μs)^30^, and *γ*_s_ = 1/(640 μs)^31^. While other methods to evaluate the sodium spin-exchange collision rate depend on estimates of the atomic collisional cross-section between Na and other species, we provide a relatively direct measurement of *γ*_s_. This value could be used, based on first principles, to calculate the actual Na–O_2_ cross-section in the mesosphere (to the best of our knowledge no experimental measurement of the Na–O_2_ spin-exchange cross-section at mesospheric conditions has been reported).

The discrepancy between collision rates estimates may be due to bias in the assumed cross-sections, large magnetic field gradients, and/or uncertainty in the sodium profile. In order to identify the reason for this discrepancy, quantitative measurements, development of an improved collision model, and parallel sodium profile measurements with lidar could be used.

We have demonstrated a method of remote magnetic field measurements in the mesosphere using a laser beam with intensity modulation at the Larmor frequency of sodium, achieving an accuracy of 0.28 mG Hz^−1/2^. This work contributes to several efforts in the scientific community to develop techniques for remote sensing of magnetic fields in the atmosphere^12,32,33^. We note that the setup used in this experiment can, in principle, be realized with components such as laser sources, modulators, and telescopes currently available commercially. Our observations show good agreement with the predictions of the geomagnetic field from the World Magnetic Model for altitudes between 85 and 100 km, and could provide input data for future assessments of this model. Further improvement of the method is possible. For instance, with laser power high enough to saturate the resonant velocity class, one can expand the beam to increase the total number of interrogated atoms. Furthermore, observing the magneto-optical resonance at short vertical sections of the elongated fluorescent column in the mesosphere can reduce the effect of broadening due to magnetic-field gradients. We found that the magneto-optical resonant signal contains broad and narrow features that depend on specific kinds of atomic collisions. The method presented in this work shows that atomic collision rates can be inferred from the observed resonances, suggesting another important application of this approach: remote sensing of collisional processes in the mesosphere.

## Methods

### Description of the model

The numerical simulation of the sodium fluorescence employs a semiclassical density-matrix model with a discretized atomic-velocity distribution, described in more detail in ref. ^26^ The evolution of the density matrix *ρ* is given by the Liouville equation:

3 $$\frac{{\mathrm{d}}}{{{\mathrm{d}}t}}\rho = \frac{1}{{i\hbar }}[H,\rho ] + {\mathrm{\Lambda }}(\rho ) + \beta .$$

The atomic level structure and the interaction with the light electric field and the geomagnetic field are described by the total Hamiltonian *H*. The term Λ contains phenomenological terms added to account for relaxation processes not described by the Hamiltonian, and *β* contains corresponding repopulation terms. Here these are spontaneous decay (omitted from the Hamiltonian due to the semiclassical approximation), collisional spin relaxation (S-damping)^34^, and the entrance and exit of atoms from the light beam due to motion of the atoms and the beam (transit relaxation), as well as velocity-changing collisions and atomic recoil, which couple the separate density matrices written for each velocity group. An effective relaxation rate for optical coherences that simulates a laser spectrum with non-negligible bandwidth is also included.

A simple model for velocity-changing collisions is used in which the velocity of the colliding atom is rethermalized in a Maxwellian distribution, independent of the initial velocity of the atom. A more realistic model would include correlation between the initial and final velocities^35^.

Equation (3) supplies a linear system of differential equations for the density-matrix elements, known as the optical Bloch equations. Terms oscillating at the light frequency can be removed from these equations under the rotating-wave approximation, and the equations can be further simplified in our case using an adiabatic approximation that is valid when the modulation rate of the optical field is much slower than the relaxation rate of the optical coherences of the density matrix^10^.

The fluorescent photon flux per solid angle emitted in a given direction can be found from the solution for *ρ* as the expectation value of a fluorescence operator^36^.

### Fit functions

The single Lorentzian shape is defined as:

4 $$L\left( {f;f_0,{\mathrm{\Delta }}f} \right) = \frac{1}{{1 + \frac{{4\left( {f - f_0} \right)^2}}{{{\mathrm{\Delta }}f^2}}}},$$

where *f* is the frequency of intensity modulation, *f*_0_ is the frequency offset of the single Lorentzian function and Δ*f* is the full-width at-half maximum (FWHM).

The double Lorentzian Γ used for the fit of the resonances obtained with the lock-in amplifier and with the differential counter is defined as:

5 $$\begin{array}{*{20}{l}} {\mathrm{\Gamma }} \hfill & = \hfill & {A_1\left[ {L\left( {f;f_{\mathrm{L}},{\mathrm{\Delta }}f_1} \right) - L\left( {f;f_{\mathrm{R}},{\mathrm{\Delta }}f_1} \right)} \right]} \hfill \\ {} \hfill & {} \hfill & { + A_2\left[ {L\left( {f;f_{\mathrm{L}},{\mathrm{\Delta }}f_2} \right) - L\left( {f;f_{\mathrm{R}},{\mathrm{\Delta }}f_2} \right)} \right]} \hfill \\ {} \hfill & {} \hfill & { + mf + g,} \hfill \end{array}$$

where *A*_1_ and *A*_2_ are the amplitudes of each Lorentzian, *f*_L_ and *f*_R_ are the offsets of the central peaks with respect to the Larmor frequency such that *f*_Larmor_ = (*f*_L_ + *f*_R_)/2 and the excursion *δf* = (*f*_R_ − *f*_L_)/2, Δ*f*_1_ and Δ*f*_2_ are the FWHM of each Lorentzian, *m* is a curve slope parameter, and *g* is a total offset parameter. Equation (5) was also assumed for the fit of the model curves shown in Fig. 6.

For the resonance obtained with the photon counter, the average flux ratio for each frequency step *f*_*i*_ of the scan was calculated as:

6 $$F_{\mathrm{R}}\left( {f_i} \right) = \frac{1}{n}\mathop {\sum}\limits_{k = 0}^{n - 1} \frac{{F_{2k}}}{{F_{2k + 1}}},$$

where *k* is an integer corresponding to every cycle of the square-wave modulation signal, and *F*_2*k*_ and *F*_2*k* + 1_ denote the photon flux in alternating sub-periods at which the laser was modulated at ± *δf*, respectively. The number of averaged point for each frequency step was *n* = 300.

Provided that the excursion *δf* is larger than the true resonance width, we assume that data can be fitted with the following function:

7 $${\mathrm{\Gamma }}_{\mathrm{R}} = \frac{{1 + A_1L\left( {f;f_{{\mathrm{Larmor}}} + \delta f,{\mathrm{\Delta }}f_1} \right) + A_2L\left( {f;f_{{\mathrm{Larmor}}} + \delta f,{\mathrm{\Delta }}f_2} \right)}}{{1 + A_1L\left( {f;f_{{\mathrm{Larmor}}} - \delta f,{\mathrm{\Delta }}f_1} \right) + A_2L\left( {f;f_{{\mathrm{Larmor}}} - \delta f,{\mathrm{\Delta }}f_2} \right)}},$$

where *A*_1_, *A*_2_, Δ*f*_1_, and Δ*f*_2_ are defined as in Eq. (5) and *f*_Larmor_ is the Larmor frequency as a free parameter.

### Noise analysis

The reported value of magnetometry noise floor was calculated based on data of a single observation run that is shown in Fig. 5a. Data were obtained with a lock-in amplifier with a time constant of *τ*_c_ = 300 ms, providing an equivalent noise bandwidth (ENBW) for the magnetometer of ENBW = 1/(8*τ*_c_) = 0.4 Hz.

The amplitude was normalized to a range between −1 and +1 and the double Lorentzian function defined in Eq. (5) was used to fit the data. The fit residuals were obtained and used as a sample that characterizes the noise of the measurements. The amplitude spectral density (ASD) of the residuals was calculated as it is shown in Supplementary Fig. 2. The average value of the ASD was calculated as $\overline {{\mathrm{ASD}}} = 0.064\,{\mathrm{Hz}}^{ - 1/2}$.

In order to obtain the average ASD in magnetic field units, a conversion from normalized amplitude to magnetic field is applied. The conversion is calculated based on a true resonance signal which is assumed to be the function fit of Eq. (5). The sensitivity to magnetic field fluctuation depends on the slope region of the resonance. Assuming the true resonance as the fitted curved Γ, a small change in the Larmor frequency will produce a small change in amplitude that we calculate as the derivative dΓ/d*f* of the fit function Γ. Conversely, a small variation in the Larmor frequency can be detected by an observed amplitude change given by (dΓ/d*f*)^−1^.

A small variation in the magnetic field yields a change in the Larmor frequency given by:

8 $${\mathrm{\Delta }}f_{{\mathrm{Larmor}}} = \gamma {\mathrm{\Delta }}B,$$

where *γ* = 699,812 Hz G^−1^ is the gyromagnetic ratio of ground-state sodium. Therefore, the sensitivity function *S*(*f*) can be obtained as:

9 $$S(f) = \frac{1}{\gamma }\left| {\frac{{{\mathrm{d}}{\mathrm{\Gamma }}}}{{{\mathrm{d}}f}}} \right|^{ - 1}$$

The absolute value is used for convenience to avoid negative sensitivity values on negative slopes of the curve (opposite response polarity). The sensitivity function is expressed in Gauss and the noise floor at each point of the resonance curve can be calculated as:

10 $$N(f) = S(f) \times \overline {{\mathrm{ASD}}} .$$

The resultant noise floor obtained from Eq. (10) is shown in Fig. 5b.

### Code availability

The simulation is based on the open-source AtomicDensityMatrix and LGSBloch packages for Mathematica, developed by two of the authors (S.R. and R.H.), and available at http://rochesterscientific.com/ADM/. The calculations in this work used a custom numerical solver, not publicly available, based on the SUNDIALS suite (https://computation.llnl.gov/projects/sundials).

## Electronic supplementary material

Supplementary Information

## Data Availability

The data that support the findings of this study may be available from the corresponding author upon request.
