# Supplementary material for: Remote sensing of geomagnetic fields and atomic collisions in the mesosphere
Source: Nat Commun. 2018 Sep 28;9:3981. doi: 10.1038/s41467-018-06396-7 (PMC6162266; doi:10.1038/s41467-018-06396-7)
Supplement: Supplementary file 1 — Supplementary Information [file 41467_2018_6396_MOESM1_ESM.pdf]

Supplementary information

Remote sensing of geomagnetic fields and atomic collisions in the mesosphere

Pedreros Bustos et al.

Supplementary figures

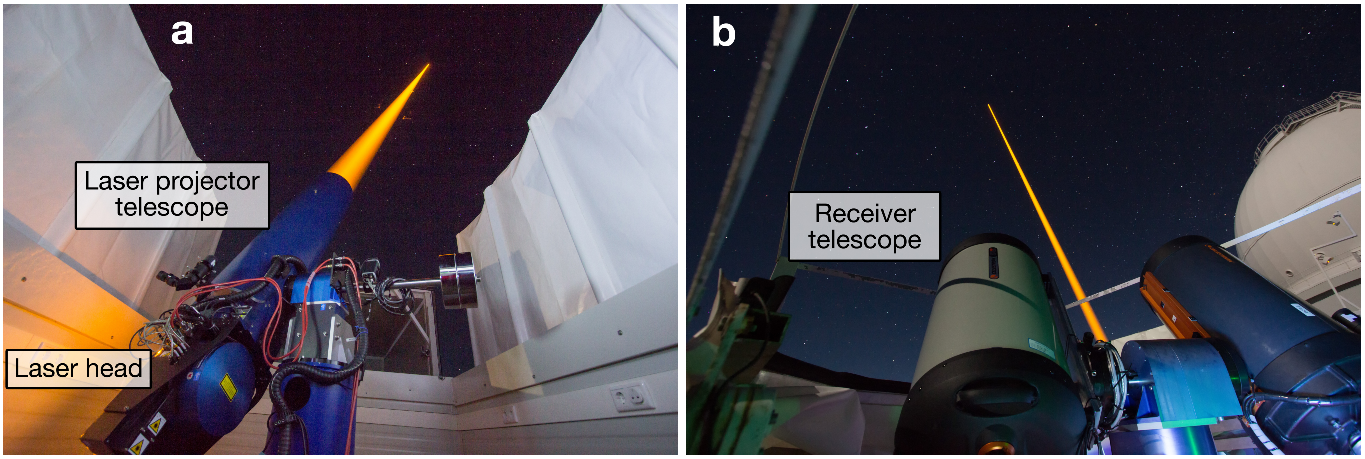

Supplementary Figure 1: Laser projector and receiver telescopes. (a) The 589 nm 20-W laser projector of the European Southern Observatory laser guide star system with the Raman fiber amplifier and laser head attached. (b) The receiver telescope was placed 8 meters away from the laser projector for detection of fluorescence from mesospheric sodium.

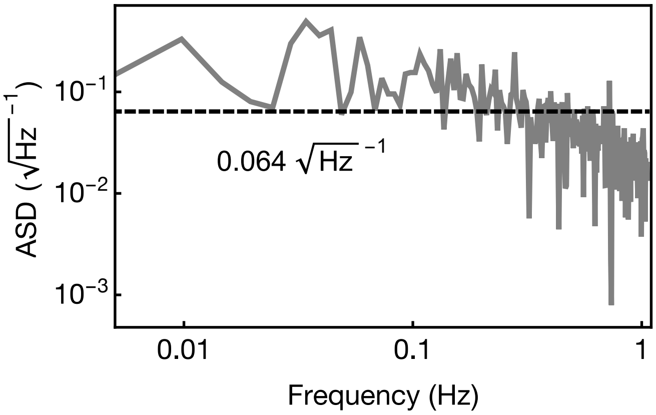

Supplementary Figure 2: Amplitude Spectral Density (ASD). The ASD of the residuals from the fit of the resonance shown in Fig. 5 was used to estimate the noise floor of the measurement. The average ASD of 0.064 $\text{Hz}^{-1/2}$ indicated by the dashed line is near the shot-noise sensitivity limit.
